# The relationship between thyroid and human-associated microbiota: A systematic review of reviews

**DOI:** 10.1007/s11154-023-09839-9

**Published:** 2023-10-12

**Authors:** Camilla Virili, Ilaria Stramazzo, Maria Flavia Bagaglini, Anna Lucia Carretti, Silvia Capriello, Francesco Romanelli, Pierpaolo Trimboli, Marco Centanni

**Affiliations:** 1 Department of Medico-Surgical Sciences and Biotechnologies, Sapienza” University of Rome, Corso Della Repubblica 79, Latina, Italy; 2 Endocrinology Unit, Santa Maria Goretti Hospital, Latina, Italy; 3 Department of Experimental Medicine, Sapienza” University of Rome, Rome, Italy; 4 Clinic for Endocrinology and Diabetology, Lugano Regional Hospital, Ente Ospedaliero Cantonale, Lugano, Switzerland; 5 Faculty of Biomedical Sciences, Università Della Svizzera Italiana (USI), Lugano, Switzerland

**Keywords:** Hypothyroidism, Hyperthyroidism, Thyroid autoimmunity, Thyroid cancer, Microbiota, Probiotics

## Abstract

In recent years, a growing number of studies have examined the relationship between thyroid pathophysiology and intestinal microbiota composition. The reciprocal influence between these two entities has been proven so extensive that some authors coined the term "gut-thyroid axis". However, since some paper**s** reported conflicting results, several aspects of this correlation need to be clarified. This systematic review was conceived to achieve more robust information about: 1)the characteristics of gut microbiota composition in patients with the more common morphological, functional and autoimmune disorders of the thyroid; 2)the influence of gut microbial composition on micronutrients that are essential for the maintenance of thyroid homeostasis; 3)the effect of probiotics, prebiotics and synbiotics, some of the most popular over-the-counter products, on thyroid balance; 4)the opportunity to use specific dietary advice. The literature evaluation was made by three authors independently. A five steps strategy was a priori adopted. After duplicates removal, 1106 records were initially found and 38 reviews were finally included in the analysis. The systematic reviews of reviews found that: 1) some significant variations characterize the gut microbiota composition in patients with thyroid disorders. However, geographical clustering of most of the studies prevents drawing definitive conclusions on this topic; 2) the available knowledge about the effect of probiotics and synbiotics are not strong enough to suggest the routine use of these compounds in patients with thyroid disorders; 3) specific elimination nutrition should not be routine suggested to patients, which, instead have to be checked for possible micronutrients and vitamins deficiency, often owed to gastrointestinal autoimmune comorbidities.

## Introduction

The human gastrointestinal tract hosts about 10^13^ microorganisms composed by bacteria, fungi, archaea, protozoa, and viruses, altogether known as gut microbiota (GM) [1]. This one represents about 70% of the whole microbiota associated with all the human microbial niches [2]. This set of microbes lives in the human gastrointestinal apparatus, sharing with the host organism different types of relationships, ranging from symbiosis to parasitism [3]. The composition of the microbiota differs in each part of the gastrointestinal tract, being influenced by the environmental pH, oxygen and antimicrobial concentration that lead to the maximal microbial concentration within the large intestine [4]. The maintenance of intestinal barrier integrity, the digestion of nutrients, the metabolism of several drugs, as well as a pivotal role in immune system development and functioning, represent the main functions of gut microbiota [5]. Such a prominent role on whole body homeostasis is so extensive that gut microbiota has been defined as a “hidden organ” that makes the set of human being and its microbiota a “superorganism” [6].

The composition of GM is strongly influenced by environmental determinants and individual behavior, beyond the shaping due to genetic background [7]. It tends to remain stable during adult life due to its resistance, also being able to come back to its initial composition due to its resilience. GM composition may even adapt to new conditions in the case of long-term perturbations; however, when the perturbations are substantial a dysbiotic state may ensue [8]. Despite the difficulties in defining a healthy composition of the microbiota, dysbiosis can be defined as an imbalance in the composition of the microbiota in favor of pathogenic species to the detriment of symbionts and commensals [8]. Several systemic and organ-specific disorders have been related to the presence of a dysbiotic state, being the evidence stronger for metabolic diseases, allergy, autoimmunity, central nervous system disturbances as well as for several types of cancer [9].

A link between GM and thyroid homeostasis has been hypothesized more than one century ago by the surgeon Harries [10]. In the following years, studies on this topic were limited due to the issues in cultivation and identification of the different bacterial strains. More recently, sequencing techniques and high-performance technologies increased exponentially the analysis of the composition of the human-associated microbiota in thyroid disorders. A number of original papers and narrative as well as systematic reviews analyzed the composition of GM in patients with different thyroid disorders. Furthermore, some papers examined the relationship between gut microbiota and the micronutrients related to thyroid homeostasis, also evaluating the effect of microbiota modulation through pro- and prebiotics administration.

The present review is aimed at answering the following specific questions:—are there variations of GM in patients with thyroid autoimmune disorders and with thyroid cancers as compared to healthy subjects?—is there evidence of a causal relationship between variations in microbiota composition and thyroid disorders?—is there an impact of gut microbiota composition on micronutrients related to thyroid homeostasis?—should a specific diet be suggested to patients with thyroid disorders to modulate their GM?—is there a rationale for the routine use of probiotics, prebiotics or synbiotics in unselected patients with thyroid disorders? To answer these questions we designed a systematic review of reviews on these topics, we synthesized the conclusions of each review included, and we discussed the summary of the main findings.

## Methods and material

### Review conduction

The present systematic review was performed following the methodology proposed by Aromataris et al. [11].

### Search strategy

The literature was searched by three authors independently (C.V., I.S. and M.C.). A 5-step search strategy was a priori adopted:

1) sentinel studies were sought in PubMed using multiple combinations of the following keywords: thyroid, Hashimoto’s thyroiditis, Graves’ disease, ophthalmopathy, goiter, thyroid carcinoma, gut microbiota, microbiome, probiotics, prebiotics, synbiotic; 2) keywords and MeSH terms were identified in PubMed; 3) PubMed, Web of Science and Scopus were searched; 4) narrative reviews, systematic reviews and meta-analyses potentially eligible were identified; 5) reviews focused on the relationship between thyroid homeostasis and gut microbiota composition were directly included in the study, while those focusing on different endocrine glands were screened and included only when the section dedicated to thyroid disorders was significant. A beginning date limit was not used, and the search was updated until July 13th, 2023. A language restriction was not applied to the research. To find possible additional studies extending the search, the reference list of the selected papers was also examined.

### Data extraction

For each article included in the present analysis the following information was extracted by three authors (C.V., M.C., I.S.) independently: authors, country, date of publication, journal, type of review (i.e., narrative review, systematic review, systematic review with meta-analysis), context of the review (i.e., focusing on specific thyroid pathophysiology aspects or on endocrinology in general), aim of the review, conclusions of the authors.

## Results

### Reviews retrieved

**Fig. 1:**
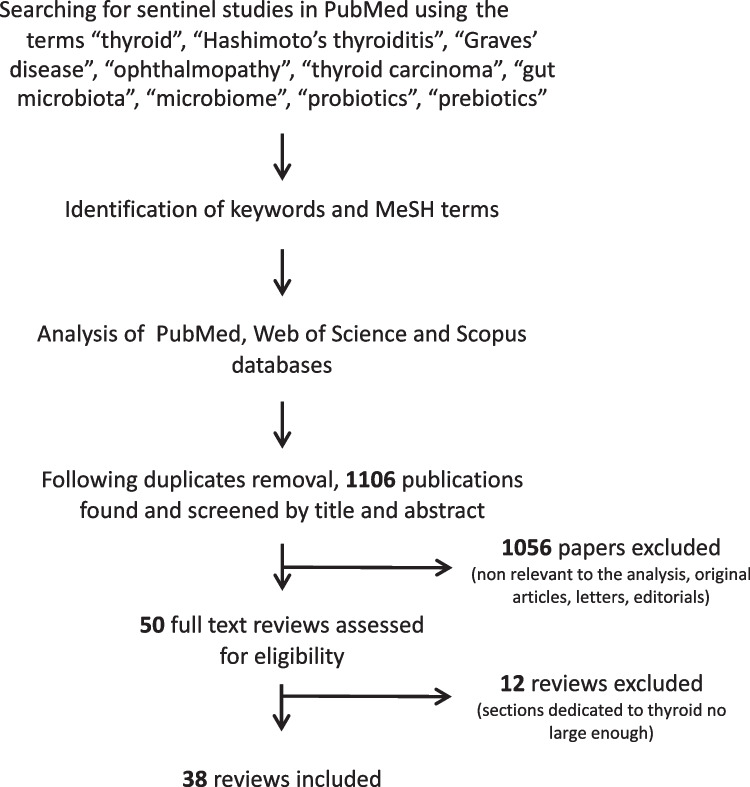
Search strategy and reviews’ selection

Applying the above search strategy, 1880 records were initially obtained. Once excluded the 774 duplicates, we analyzed 1106 papers; thirty-eight of them have been included [12–49] in the analysis since the others were excluded because they were not review articles, were not relevant to the analysis or did not contain a large section dealing with the above topics. In Fig. 1 is reported the search strategy and the flow of articles.

The reviews included in our analysis were published in the last twelve years (2012–2023). Both objectives and conclusions of the 38 reviews were clearly reported. Most of the reviews included were narrative, and so the pre-defined patient-centered questions (e.g., PICOS, participants, interventions, comparators, outcomes, and study design) were not reported. Table 1 indicates the main features of the reviews contained in the present systematic review.

**Table 1:** List of the reviews included in the present systematic review

**FIRST AUTHOR**	**COUNTRY**	**DATE OF PUBLICATION**	**JOURNAL**	**TYPE OF REVIEW**	**TOPIC (thyroid/endocrinology)**
Mori [12]	Japan	27 November 2012	Discovery Medicine	NR	Hashimoto’s Thyroiditis
Kunc [13]	Poland	26 October 2015	Acta Biochimica Polonica	NR	Endocrinology
Covelli [14]	UK, Italy	7 January 2017	Journal of Endocrinological Investigation	NR	Graves’ Disease and Orbitopathy
Virili [15]	Italy	4 February 2017	Molecular and Cellular Endocrinology	NR	Thyroid hormone metabolism
Köhling [16]	UK, Germany	6 July 2017	Clinical Imuunology	NR	Thyroid Autoimmunity
Virili [17]	Italy	8 October 2018	Reviews in Endocrine & Metabolic Disorders	NR	Hashimoto’s Thyroiditis
Fröhlich [18]	Germany	27 June 2019	Trends in Endocrinology & Metabolism	NR	Thyroid Disorders
Ihnatowicz [19]	Poland	2 October 2019	Annals of Agricultural and Environmental Medicine	NR	Hashimoto’s Thyroiditis
Ejtahed [20]	Iran	2020	Endocrine, Metabolic & Immune Disorders—Drug Targets	NR	Thyroid Disorders
Fenneman [21]	The Netherlands, USA	15 May 2020	Biochemical Society Transactions	NR	Endocrinology
Knezevic [22]	Austria	12 June 2020	Nutrients	NR	Micronutrients pivotal in thyroid homeostasis
Masetti [23]	UK	5 November 2020	European Thyroid Journal	NR	Graves’ Disease and Orbitopathy
Opazo [24]	Chile, Belgium	23 November 2020	Critical reviews in food Science and Nutrition	NR	Micronutrients pivotal in thyroid homeostasis
Docimo [25]	Italy	4 December 2020	Frontiers in Endocrinology	NR	Thyroid Diseases
Virili [26]	Italy	17 February 2021	Best Practice & Research Clinical Endocrinology & Metabolism	NR	Thyroid Autoimmunity
Qi [27]	China	15 March 2021	Gut Microbes	NR	Endocrinology
Sturov [28]	Russia	May 2021	Archiv Euromedica	NR	Thyroid autoimmunity
Fernàndez-Garcìa [29]	Spain	25 May 2021	Molecular and Cellular Endocrinology	NR	Thyroid autoimmunity
Ferreira [30]	Brazil	4 June 2021	Frontiers in Nutrition	NR	Micronutrients pivotal in thyroid homeostasis
Bargiel [31]	Poland	16 August 2021	Journal of clinical medicine	NR	Thyroid Dysfunction
Cao [32]	China	17 November 2021	Graefe’s Archive for Clinical and Experimental Ophtalmology	NR	Graves’ Orbitopathy
Gong [33]	China	17 November 2021	Frontiers in Endocrinology	SR + MA	Thyroid Autoimmunity
Hou [34]	China, Canada	22 December 2021	Frontiers in Cellular and Infection Microbiology	NR	Graves’ Disease and Orbitopathy
Bogulawska [35]	Poland	1 January 2022	European Thyroid Journal	NR	Thyroid Autoimmunity
Zhou [36]	China	4 January 2022	Frontiers in Cell and Developmental Biology	NR	Graves’ Disease and Orbitopathy
Wang [37]	China	5 January 2022	Frontiers in Endocrinology	NR	Graves’ Orbitopathy
Liu [38]	China	16 February 2022	Frontiers in Molecular Bioscience	NR	Graves’ Disease and Orbitopathy
Danailova [39]	Bulgaria	5 April 2022	International Journal of Molecular Science	NR	Hashimoto’s Thyroiditis
Liu [40]	China	27 May 2022	Cancers	NR	Thyroid Cancer
Jiang [41]	China	18 August 2022	Frontiers in Endocrinology	NR	Thyroid Diseases
Belvoncikova [42]	Slovakia	14 September 2022	International Journal of Molecular Science	NR	Thyroid Autoimmunity
Calcaterra [43]	Italy	23 September 2022	Minerva Pediatrics	NR	Thyroid Autoimmunity
Sawicka-Gutaj [44]	Poland, Italy	3 November 2022	International Journal of Molecular Science	SR + MA	Thyroid Autoimmunity
Wu [45]	China	18 December 2022	Microbiological Research	NR	Endocrinology
Macvanin [46]	Serbia, Saudi Arabia	4 January 2023	Frontiers in Endocrinology	NR	Thyroid Diseases
Fenneman [47]	The Netherlands	6 January 2023	Thyroid	NR	Thyroid hormone metabolism/ Thyroid Autoimmunity
Stramazzo [48]	Italy	28 March 2023	Advances in experimental medicine and biology	SR	Thyroid Diseases
Zawadza [49]	Poland, Iran	16 May 2023	Annals of Agricultural and Environmental Medicine	SR + MA	Probiotic use in Thyroid Diseases

### Findings of the reviews included

The aim and the main conclusions of the thirty-eight reviews were schematically reported in Table 2. In brief, these studies analyzed the GM composition in patients with thyroid autoimmune disorders, with or without functional impairment as well as in those with thyroid cancer. Some of the included reviews reported the results of studies dealing with thyroid diseases and microbiota belonging to other biological niches such as the oral or thyroidal (tumoral and peritumoral) ones. Moreover, the bidirectional relationship between microbiota composition and thyroid-related micronutrients intake has been examined. Finally, the perspectives of gut microbiota modulation by probiotics, prebiotics or synbiotics, by dietary habit or by fecal microbiota transplantation have been stressed (Table 3).

**Table 2:** Aim and main conclusions of the reviews included in the present systematic review

**FIRST AUTHOR**	**AIM**	**MAIN CONCLUSIONS**
Mori [12]	To review the pathogenic significance of the gut microbiota composition in Hashimoto’s thyroiditis	Despite their limited number, some studies carried out in animal models suggested that intestinal pathogens and symbiotic microorganisms may influence extra-intestinal immune responses and may lead to loss of immune tolerance vs thyroid tissues
Kunc [13]	To evaluate the microbiome role in endocrine system’s modulation	Several metabolic steps of iodothyronines are influenced by gut microbiota
Covelli [14]	To focus on a possible link between gut microbiota, thyroid disease and orbital involvement	Some evidence suggest that the most important risk factors for the development of Graves’ disease are able to impact on gut microbiota composition
Virili [15]	To review the knowledge focused on the interactions of gut microbiota with thyroid-related micronutrients and its involvement in metabolic steps of iodothyronines	Iodothyronines binding, uptake, deconjugation and deiodination are all abilities of gut bacterial content. The net effect of dysbiosis in this frame is not known
Köhling [16]	To review the available evidence on bacterial involvement in Graves’ disease pathogenesis	The link between gut microbiota composition and Graves’ disease onset or progress has not elucidate yet
Virili [17]	To focus on the features characterizing the reciprocal influence between gut microbiota composition and thyroid autoimmunity described in the literature	Most of the evidence about a link between microbiota composition and Hashimoto’s thyroiditis emerges from studies in animal models. The studies on human beings are few, carried out in a unique country and do not involve enough patients to draw conclusions
Fröhlich [18]	To summarize the knowledge about the multiple interferences between thyroid pathophysiology and gut microbiota composition	An altered composition of gut microbiota has been clearly linked to thyroid autoimmunity, even if a possible causative role of dysbiosis in triggering thyroid autoimmunity is still under debate. Microbiota composition may also impact on micronutrients absorption and thyroid hormone recycling
Ihnatowicz [19]	To examine the role of nutritional factors in Hashimoto’s thyroiditis management with a focus on gut microbiota alterations and the possible nutritional impact on its composition	Hashimoto’s thyroiditis patients, hypothyroid or not, shows peculiar features of gut microbiota that may be shaped by consumption of specific foods
Ejtahed [20]	To discuss the main features of gut microbial composition in functional and morphological alterations of thyroid gland	Evidence supports bidirectional associations between thyroid diseases and intestinal flora composition. The hypothesis of a causative role of dysbiosis in thyroid disorders deserves more studies
Fenneman [21]	To provide insights into the possible role of gut microbiota and its metabolites in the pathophysiology of Hashimoto’s thyroiditis and type1 diabetes	The gut microbiota composition seems to be related to Hashimoto’s thyroiditis progression. Further studies are needed to ascertain a microflora role in the development of this disease
Knezevic [22]	To examine the interplay between gut microbiota and thyroid disorders with a specific focus on micronutrients crucial for thyroid gland homeostasis	Accumulating data witness the existence of a thyroid-gut axis, linking the effect of gut bacteria on the immune system, thyroid function and absorption of micronutrients essential for thyroid homeostasis maintenance
Masetti [23]	To summarize the evidence of microbiome involvement in the pathogenesis of Graves’ disease and orbitopathy	Only little evidence has been attained about the effect of microbial composition, in different body’s niches, in contributing to thyroid autoimmunity
Opazo [24]	To analyze the different aspects of iodine nutrition and its biological role in thyroid hormones balance and in shaping gut microbiota	Iodine intake may affect the composition and diversity of intestinal microbiota. Both thyroid autoimmunity and dysbiosis are linked to iodine intake
Docimo [25]	To characterize the gut microbiota in autoimmune thyroid disorders, evaluating the impact of dysbiosis on their treatment and prognosis. A specific para is dedicated to benign thyroid nodules and papillary thyroid cancer pathophysiology and progression	A change in quality and quantity of intestinal microbes is associated to thyroid autoimmune disorders as well as to thyroid carcinoma
Virili [26]	To assess the knowledge about the gut microbiota features in patients with thyroid autoimmune diseases and the reciprocal interactions between the microflora and the most common treatments used for thyroid diseases	The linkage between microbiota composition and thyroid autoimmunity has been proven in different models; evidence are more strong for Graves’ diseases than for Hashimoto’s thyroiditis due to the different functional status of patients enrolled in the studies
Qi [27]	To analyze the impact of the gut microbiota composition on the reproductive and metabolic endocrine system examining the role of gut microflora on thyroid disorders	Thyroid cancer, hypo- and hyperthyroidism are characterized by peculiar microbiota composition. The uptake of iodine, selenium iron and zinc may be affected by gut microbiota composition
Sturov [28]	To consider the interaction between gut microbiota and the development of thyroid autoimmunity and the characteristics of microflora composition in patients with thyroid dysfunction	Changes in microbiota profile is one of the factors involved in the development of thyroid autoimmunity
Fernàndez-Garcìa [29]	To review the principal known interactions between thyroid, gut microbiota and immune system	Several mechanisms controlling thyroid homeostasis may be affected by gut microbiota composition
Ferreira [30]	To sum up the knowledge about the relationship between gut microflora and selenium status with a focus on bioavailability of selenocompounds and thyroid disorders	Specific bacterial strains belonging to gut microbiota (e. g. belonging to Lactobacillus genus) are able to provide more bioavailable forms of selenium and microbiota may impact on enzymatic activity of selenoproteins
Bargiel [31]	To describe the evidence about the association between microbiota and its metabolites to thyroid dysfunction and autoimmunity	The microbiome is related to thyroid malfunctioning but if dysbiosis is a cause or an effect of thyroid disorders is not known
Cao [32]	To examine the literature about risk factors for Graves’ ophthalmopathy	A link between gut microbiota composition and Graves’ ophthalmopathy has been described but it is unclear whether the disease might be controlled regulating flora composition
Gong [33]	To assess the differences in fecal microbiota composition between 160 healthy controls and 196 patients with thyroid autoimmune disorders meta-analyzing 8 studies	An association between autoimmune thyroid disorders and an altered microbiota composition has been found at family, species and genera level
Hou [34]	To discuss the changes and potential effect of the gut microbiota in the pathogenesis of GD and GO and comment on possible therapies targeting gut microbiome	Significant differences characterize GD/GO patients from healthy controls due to the reduced microbial diversity and the increased concentration of *Lactobacillus*, *Prevotella* and *Veillonella*
Bogulawska [35]	To present the up-to-date knowledge about molecular and cellular mechanisms underlying the pathology of autoimmune thyroid disorders with a specific focus on gut microbiome	In the literature there are several evidence demonstrating that there is a link between autoimmune thyroid disorders and microbiome but the actual functional consequences of microbiome alteration require more extensive research
Zhou [36]	To evaluate the knowledge about the role of genetic background, epigenetics, cellular immunology and gut microbiota in the occurrence of Graves’ disease	Opposite results have been published about the gut microbiota composition at phyla level in patients with GD due to small sample sizes and the different regions in which the studies have been carried on. However, at genus level, some results seem to be more consistent, such as the higher abundance of *Lactobacillus* and* Prevotella*
Wang [37]	To review the last evidence about gut microbiome involvement in the pathogenesis of Graves’ ophthalmopathy	The induction of thyroid autoimmunity in two animal models of GD and GO revealed a different clinical response also related to a different gut microbial composition A different gut microflora composition has been described in patients with GO as compared to both GD and healthy subjects
Liu [38]	To give a synopsis of the correlation between gut microbiota composition, with a specific focus on microbial metabolites, and Graves’ disease	Metabolomic analysis described some differences in metabolic pathways between Graves’ disease patients and healthy subjects but their consequences on thyroid homeostasis is still under investigation
Danailova [39]	To evaluate the effect of microbiota in development and exacerbation of Hashimoto’s thyroiditis with a focus on its possible nutritional management	An appropriate dietary regimen, based on an anti-inflammatory diet, may provide an optimal nutrition in Hashimoto’s thyroiditis patients
Liu [40]	To evaluate the relationship between gut microbiota, thyroid function variations and thyroid cancer	Gut microbiota is significantly different in patients with thyroid cancer as compared to healthy subjects. The analysis of tumoral specimens revealed different microbiota composition in peritumoral and tumoral tissues, with specific variations related to tumor subtype, metastases presence and patient’s age
Jiang [41]	To analyze the variations in gut microflora composition and metabolites in autoimmune, functional and tumoral thyroid disorders	Several aspects of thyroid homeostasis might be related with gut microbiota composition but the evidence about the causative role of microflora on thyroid disorders are still lacking
Belvoncikova [42]	To summarize the results of the studies dealing with fecal microbiota transplantation experiments in thyroid autoimmune disorders field	The different experiments strongly suggested the involvement of gut microflora on thyroid autoimmunity pathogenesis
Calcaterra [43]	To describe the reciprocal influence between gut microbiota, thyroid hormone metabolism and thyroid autoimmunity with a focus on probiotics use in a pediatric setting	Deepening in understanding the connections between gut microbiota, thyroid hormones metabolism and thyroid-related micronutrients would implement microbiota-targeted therapies in thyroid disorders also in a pediatric perspective
Sawicka-Gutaj [44]	To clarify if microbiota composition is altered in patients with thyroid autoimmunity	Significant alteration of the diversity indexes and overall composition have been described in autoimmune thyroid disorders. While higher diversity has been detected in patients with Hashimoto’s thyroiditis, lower diversity has been described in patients with Graves’ disease, with a higher relative abundance of Bacteroidetes and Actinobacteria
Wu [45]	To sum up the knowledge about gut microflora characteristics in patients with autoimmune and tumoral thyroid disorders	More clinical research are needed to estimate the possible causative role on thyroid disorders of gut dysbiosis and the effects of its modulation
Macvanin [46]	To review novel findings on the connection between thyroid and gut microbiome with a specific focus on probiotics with antioxidant properties on thyroid disorders	Despite the promising potential in improvement of thyroid function of probiotic supplementation, further human studies are needed
Fenneman [47]	To address the main features of thyroid-gut axis	Dysbiotic state characterizes autoimmunity but the proof of a causative role is still lacking. Recent papers on microbiota transplantation in murine models suggest that it might represent a therapeutic tool which deserves further evaluation
Stramazzo [48]	To analyze the more recent advancements in the relationship between gut microflora composition and thyroid autoimmune and non-autoimmune disorders, extending the analysis to the microbiota resident in other biological niches	The results of the studies examined strengthen the bidirectional relationship between the intestine with its microbial set and thyroid homeostasis supporting the existence of the thyroid-axis
Zawadza [49]	To evaluate the efficacy of probiotics, prebiotics or synbiotics supplementation in primary hypothyroidism	The results of the only two RCT published on the topic, involving 136 patients, suggested that routine administration of probiotics, prebiotics or synbiotics may result in little to not benefit in uselected patients with primary hypothyroidism

**Table 3:** Summary of findings in the present systematic review

**Question of the present systematic review**	**Conclusion**	**References supporting these findings**
Is there a variation in microbiota composition in patients with Hashimoto’s thyroiditis?	Gut microbiota composition significantly differs from healthy controls in terms of higher richness and significant α-diversity. Variations has been detected in relative abundance at phylum, family, genus and specie levels. Some phyla and genus significantly correlated with TSH level as well as with anti-TPO and anti-Tg autoantibodies. However, the geographical clustering of the studies imposes a deepening in the topic	[12, 17, 19–21, 26, 28, 29, 31, 34, 35, 39, 42–44, 47, 48]
Is there a variation in microbiota composition in patients with Graves’ disease and Graves’ ophthalmopathy?	Most of the available evidence, derived from studies prevalently conducted in China, agrees in describing a decrease of all indices of richness and diversity and of the Firmicutes/Bacteroidetes ratio in patients with GD and GO. The results from a European study seem to be significantly different. Even more heterogeneous are the results regarding lower taxonomic levels	[14, 16, 23, 26, 32–34, 36–38, 41, 43, 44, 48]
Is there a variation in microbiota composition in patients with thyroid cancer?	The very few studies available seem to indicate the reduction of the butyrate-producing gut microbiota as potential signature of thyroid cancer. The same SCFA by inhibiting histone deacetylase, seems to activate NIS re-expression in thyroid cancer cells, inducing iodine uptake and redifferentiation	[20, 22, 25, 40, 41, 45, 48]
Is there an impact of gut microbiota composition in micronutrients related to thyroid homeostasis?	The link between micronutrients homeostasis and gut microbiota composition is complex and bidirectional. Iodine may alter gut microbiota composition, affecting both resident and pathogen bacteria due to its intrinsic antimicrobial activity. A competition for selenium uptake between resident microflora and the host emerges in condition of limited selenium availability. Similarly, gut microbiota and the host compete for iron absorption. An excess of iron intake may favor the increase of intestinal pathogens	[15, 18, 19, 22, 24, 26, 27, 29–32, 34, 39, 46]
Is there a rationale for the use of probiotics, prebiotics or synbiotic in unselected patients with thyroid disorders?	There are no evidence supporting the routine use of probiotics, prebiotics or synbiotic in unselected patients with thyroid disorders	[13, 15, 18, 20, 22, 23, 25, 26, 34, 35, 38, 40, 46, 48, 49]
Should a specific diet be suggested to patients with thyroid disorders?	Despite dietary habits represents one of the major determinant in microbiota shaping, the evidence of a specific interplay between diet, gut microbiota and thyroid disorders is still scanty. The usefulness of gluten free diet in patients with thyroid autoimmunity is, as yet, merely an hypothesis, in the absence of celiac disease or gluten intolerance. Patients should be checked for possible micronutrients and vitamins deficiency, also keeping in mind the possible coexistence of gastrointestinal autoimmune disorders	[14, 19, 34, 39]
Is there evidence of a causal relationship between variations in microbiota composition and thyroid disorders?	Overall, the evidence suggested a complex link between gut microbial composition, gut permeability and thyroid functional, immunological and morphological homeostasis but are not sufficient to establish a clear cause-effect in the pathophysiology of these disorders. However, the evidence obtained from experimental models should prompt the scientific research to further investigate the topic	[15–17, 20, 47, 48]

## Discussion

**Fig. 2:**
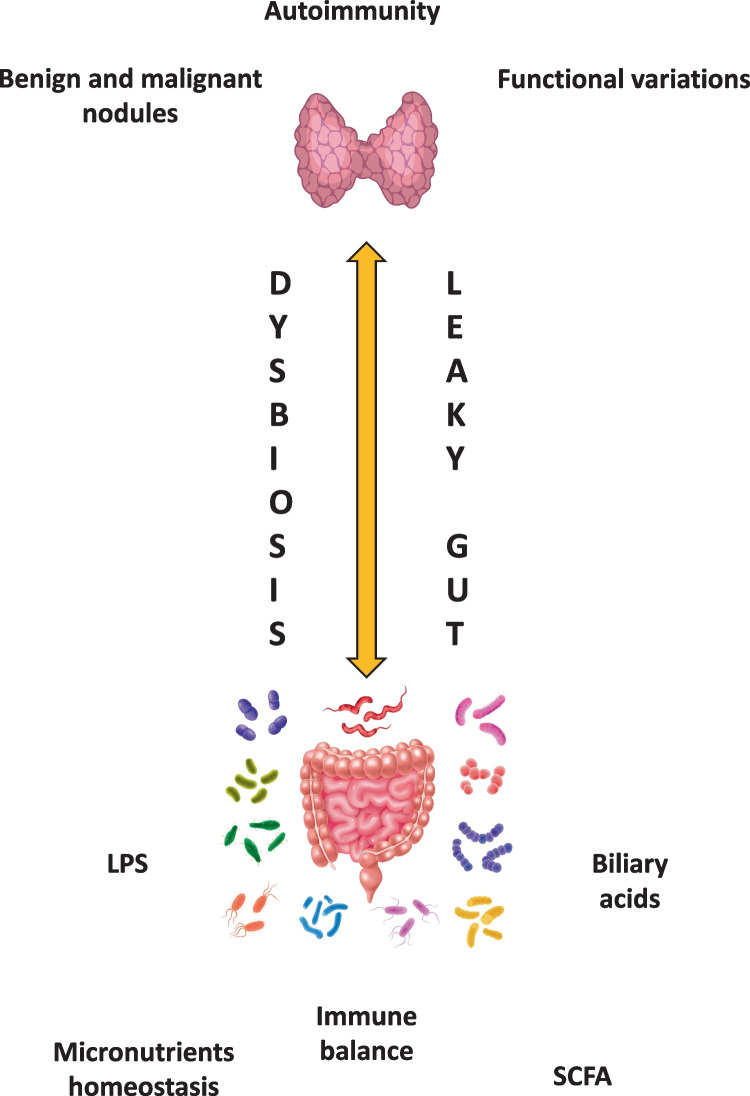
Main elements involved in the “gut-thyroid axis”

Owing to the several links between the gut, with microbial set its, and thyroid homeostasis, the term “gut-thyroid axis” has been recently proposed [50] and the principal elements involved are depicted in Fig. 2.

Thyroid hormones exert a key role in the development and differentiation of intestinal epithelium, thus actively participating in the intestinal barrier integrity. Among the intestinal effects of thyroid hormones, the more relevant are the regulation of intestinal epithelium turnover [51] and the induction of intestinal alkaline phosphatase. This latter is a brush-border enzyme that dephosphorylates the proinflammatory bacterial endotoxin lipopolysaccharide (LPS), thereby preventing its translocation into the systemic circulation [52, 53]. This process has been correlated to the induction of autoimmunity in genetically- predisposed subjects, since the intestine hosts the 70% of the immune system (Gut Associated Lymphoid Tissue – GALT) [54]. Indeed, the entry into the systemic circulation of bacterial antigens and their epitopes, even through the molecular mimicry, may trigger auto-aggressive processes [55] by a local or by-stander activation. Furthermore, it represents a potential site for the activation of autoreactive cells and initiation/propagation of autoimmune diseases, also involving organs far from the intestine [56]. To note, a condition of gut leakiness has been also related to the growth and progression of cancers involving organs other than thyroid [54].

On the side of thyroid function variations, they have also been related to a different gut microbiota composition: in hypothyroid patients an increased prevalence of small intestine bacterial overgrowth has been described [57] as well as a dysbiotic state in patients with thyroid hyperfunction [58]. Conversely, it was demonstrated that germ free (GF) mice, devoid of microorganisms colonization, have lower radioactive iodine uptake and 25% higher values of TSH than conventionally reared ones [59]. To note, a direct binding of thyroid hormones to gut bacterial strains has been firstly demonstrated in the 60’s, suggesting the intestine to be a reservoir for thyroid hormones [60]. It has been also hypothesized a role for the intestine and the associated microbiota in thyroid hormones metabolism since:—some bacterial strains possess glucuronidase and sulfatase activities, enabling thyroid hormone enterohepatic recycling;—in mice, intestinal wall possesses deiodinases isoforms and ornithine decarboxylase allowing the synthesis of thyroid hormones’ derivatives;—deiodinases activities, that have been detected in the intestinal content of rats, are inhibited by resident microbiota [17];—in animal models, LPS injection is able to modulate hepatic and pituitary deiodinases activity [13]. Thyrocytes themselves are able to respond to circulating LPS because of their expression of functional Toll-like receptor 4 (TLR4), which is able to induce both Na^+^/I symporter (NIS) and thyroglobulin (Tg) gene expression [61]. LPS is also able to decrease thyroid hormone receptor expression in hepatic extracts [13]. Interestingly, SCFAs (Short Chain Fatty Acids) (butyrate, propionate and acetate) produced by resident microbiota are able to inhibit histone deacetylase and to activate Mitogen-activated protein kinase (MAPK) pathway that may induce hyperphosphorylation and thus increased transcription of thyroid hormone receptor [13]. Some papers reported a variation in SCFA concentration in patients with different thyroid disorders [31]. Another key element of the thyroid-gut axis is represented by bile acids homeostasis. Indeed, secondary biliary acids, formed in the colon through deconjugation and dehydroxylation by colic microbiota, are able to interact with Takeda G-protein coupled receptor 5 (TGR5), a receptor that stimulates type-2 deiodinases in brown adipose tissue, increasing local triiodothyronine (T3) production. On the other hand, thyroid hormones regulate biliary acids’ metabolism increasing the liver expression of cholesterol 7α-hydroxylase (CYP7A1). Interestingly, one of the secondary bile acids, deoxycholic acid, possesses a selective antimicrobial effect due to its ability to induce bacterial membrane damage. Noticeably, the composition of primary and secondary biliary acids is significantly different in patients with hyper- or hypothyroidism [21, 31].

In the next paragraphs, we will stress the relationship between the microbiota, its byproducts and the more common thyroid disorders.

### Hashimoto’s thyroiditis and hypothyroidism

Already in the 80’s, it has been shown that GM conferred a greater susceptibility to the development of thyroiditis in rodents grown in conventional conditions compared to GF ones [62]. To note, sequences homologies have been found between thyroid-specific antigens [thyroid peroxidase (TPO) and Tg] and surface antigens of several bacteria, both pathogens or commensals, normally belonging to gut microbiota [63]. Later, it has been proven that TLR4 activation by LPS is able to trigger thyroiditis in NOD H2h4 mice [64]. Other clues of a role for GM in Hashimoto’s thyroiditis (HT) pathogenesis are related to the detection of the leakiness of the gut barrier detected in a morphologic and functional study in euthyroid patients with this disorder [65]. This evidence has been strengthened by the evidence that serum zonulin, an indirect index of increased gut permeability, is increased in HT patients [66].

Recently, a meta-analysis described the significant differences of GM composition in HT patients compared to healthy subjects. Sawicka-Gutaj et al. [44] reported that ACE and Chao1, indices describing microbial richness, and the Shannon index, reflecting the communities’ diversity, were increased in HT patients as compared to healthy controls. However, the Simpson index, which reflects the community diversity too, was lower in HT patients [44]. Overall, these results are in keeping with the longer gastrointestinal transit time that is a key sign in hypothyroid patients and that has been related to increased prevalence of small intestine bacterial overgrowth [57].

At phylum level, Bacteroidetes showed an increased relative abundance and Firmicutes a slightly reduced one compared to control subjects. To note, the Firmicutes/Bacteroidetes (F/B) ratio is known to be an indicator of normal intestinal homeostasis; this ratio’s increase or decrease has been suggested as flag of dysbiosis. [42]. A decrease in this ratio has been described in GD but in HT patients inconsistent results were reported [44].

At family level, some studies demonstrate that Lachnospiraceae, Bacteroidaceae, Enterobacteriaceae, Alcaligenaceae, Coriobacteriaceae, Erysipelotrichiaceae and Bacillobacteriaceae were increased in the gut microbiota of the HT patients; differently, Ruminococcaceae, Prevotellaceae, and Veillonellaceae were reduced [42]. Interestingly, these last two families are involved in the induction of regulatory T lymphocytes (Tregs) in the gut [42, 43].

At genus level, it was observed that *Bacteroides, Faecalibacterium, Prevotella and Lachnoclostridium* genera were lower, while *Blautia, Ruminococcus, Roseburia, Fusicatenibacter, Romboutsia, Dorea* and *Eubacterium* genera were higher in HT patient fecal samples than in healthy controls [44].

At species level, the most important result of the above meta-analysis was the increased relative abundance of *Bacteroides fragilis*, a bacterium able to activate the expression of NLR family pyrin domain containing 3 (NLRP3), an inflammasome component overexpressed in thyroid tissue of patients with HT [33]. When analyzed by Spearman’s correlation, some phylum, family and genus such as Bacteroides, Ruminococcaceae, Enterobacteriaceae, Veillonella, Streptococcus and Lactobacillus positively correlated with antithyroperoxidase antibodies (TPO) and negatively with TSH levels; moreover, the genus Streptococcus positively correlated with antithyroglobulin antibodies levels [42, 44, 47].

One study examined the gut microbiota composition in untreated patients with non-autoimmune hypothyroidism as compared to healthy subjects [67]. The authors described greater richness but lower diversity in the hypothyroid group, with increased F/B ratio and LPS serum concentration; also the SCFA producing ability was significantly reduced [67]. In the last years, it has been also evaluated the relationship between functional thyroid disorders and the composition of microbiota belonging to body niches different from the gut one [48]. A significant alpha and beta diversity on salivary samples has been described by Dong et al. [68] by comparing the microbiota of 20 healthy control (HC) and 20 subjects affected by subclinical hypothyroidism (SH). It was observed that salivary microbial composition of SH group was characterized by a major richness without the identification of a dominant species. At the phylum level, there was a similar composition between the two groups but a different distribution of 45 taxa [68].

#### Microbiota composition and levothyroxine treatment

In patients with hypothyroidism, oral levothyroxine sodium is the treatment of choice. This treatment must be personalized based on the weight and age of patients, but its efficacy depends on the absorbed hormonal fraction [26]. It has been hypothesized that levothyroxine treatment efficacy might be influenced by the composition of the gut microbiota [69]. Indeed, once absorbed at the small intestine level, thyroxine is metabolized by deiodinases but a significant fraction may be glucurono-conjugated and sulfated at liver level, rendering it more soluble in water and allowing its elimination in the intestine along with the bile [70]. It has been demonstrated that bacterial glucuronidase and sulfatase activities are able to give back thyroxine the possibility to be reabsorbed through the enterohepatic recycling [71, 72]. A recent study compared gut microbiota composition in subclinical hypothyroid patients with stable or increasing levothyroxine requirement, describing a different relative abundance at genus level in *Alistipes* and *Ruminococcus* (some strains belonging to these genera possess beta-glucuronidase activity) and in *Anaerotruncus* genus (involved in intestinal barrier stability through butyrate production) [73].

### Graves’ disease, with or without orbital involvement, and antithyroid treatment

Using animal models established by immunization with human TSHR, Masetti et al. [74] and Moshkelgosha et al. [75] analyzed how differences in gut microbiota influence the clinical manifestation of GD and Graves’ ophthalmopathy (GO) with two experiments: the first one comparing the same mouse model (BALB/c mice) placed in two different locations (Germany, UK); the second one comparing two different mice strains (C57BL/6 and BALB/c mice). Gut microbiota compositions resulted significantly different in both the experiments and correlated with clinical manifestation of GD/GO. In another experiment, Moshkelgosha et al. [76], before the TSHR immunization, administrated antibiotic vancomycin that lowered the richness and diversity of gut microbiota, also reducing F/B ratio. A significant reduction of Tregs in orbital lymph nodes and GD/GO-like clinical features has been observed. These studies suggested a crucial role of gut microbiota in the clinical manifestation of GD and GO.

In the last years a growing number of papers has faced the study of gut microbiota in patients with GD and GO. A condition of increased intestinal permeability, proved by the increase of circulating markers of leaky gut (LPS, zonulin, and D-lactate) has been described in these patients by Zheng et al. [77]. Moreover, higher LPS levels were associated with more severe hyperthyroidism, higher TSH Receptor Antibodies (TRAb) concentrations, and a worse course of both hyperthyroidism and orbitopathy [78]. A recent meta-analysis [44] examined 12 papers, mostly from China, analyzing a total of 563 patients with GD/GO who underwent fecal microbial analysis. The meta-analysis showed a clear trend toward decreasing values of all indices of richness and diversity in GD patients as compared to healthy controls. In most of the studies, the F/B ratio was lower in GD patients than in healthy individuals, suggesting the presence of dysbiosis in GD patients. Similar modifications were observed in GO patients. However, quite opposite results have been reported by Masetti et al. [23] that anticipated the results of the INDIGO study, a large-scale analysis in GD and GO patients in four European countries. In this study, fecal samples were obtained from untreated patients or within 6 weeks from treatment initiation. No significant differences emerged in alpha and beta diversity indices. Bacteroidetes were significantly decreased, while the F/B ratio was significantly higher in GD/GO than in healthy controls. These conflicting results may be explained by the different geographical origin of the patients with different environmental exposures, namely dietary habit [78]. Back to the results in GD patients from Sawicka-Gutaj et al. [44], a trend toward an increased abundance of Bacteroidetes and Actinobacteria at the phylum level, reflected in a higher abundance of *Prevotella* and *Bifidobacterium* at the genus level, has been observed. Similarly, a higher abundance of *Prevotella* was reported in GO patients.

Among clinical parameters, TRAb levels positively correlated with *Prevotella*, *Bifidobacterium* and *Lactobacillus*, while CAS (clinical activity score) was associated with *Bacteroides* abundance [79]. Noticeably, some species belonging to *Prevotella* genus, through the activation of TLR 2, are able to induce the secretion of proinflammatory cytokines and to promote neutrophil recruitment. An increased concentration of *Prevotella* genus has been described in HIV infection, obesity, hypertension and Nonalcoholic Fatty Liver Disease (NAFLD), as well [80]. Similarly, some *Bifidobacterium* and *Lactobacillus* strains could exert a pathogenic activity in autoimmune thyroid disorders (ATD) through molecular mimicry, due to their structural homology with the amino acid sequences of human TPO and Tg [81]. This evidence could explain the findings of Jiang et al. [82] and Chen et al. [83], which, analyzing the fecal microbiota in GD patients, revealed that the abundance of *Lactobacillus* was significantly higher in TPO autoantibodies (TPOAb) positive GD patients than that in TPOAb negative ones. Moreover, it could potentially justify the conflicting findings of Ishaq et al. [84] who, antithetically, described a reduction in *Bifidobacterium* and *Lactobacillus* in GD patients.

Fewer evidence is available about the effect of GD/GO therapy on microbiota and vice versa. Maier et al. [85] assessed the effect of methimazole (MMI) and of propylthiouracil (PTU) on 40 selected bacterial strains *in vitro*, finding minimal influence. On the contrary, Chen et al. [83] analyzed GM in MMI treated GD patients reporting a significantly improved diversity after 3–5 months treatment and a significant reduction of *Lactobacillus*. Sun et al. [86] compared microbiota modifications in GD patients treated with MMI or PTU. The MMI group showed more Firmicutes at the phylum level, while the PTU group was characterized by higher abundance of Bacteroidetes. The microbial dysbiosis index (MDI) and the F/B ratio suggested that dysbiosis occurred in both drug-treated groups. Interestingly, GD treatment reduced some SCFA-producing bacteria. It has been reported that, upon PTU treatment, a higher percentage of patients had subtherapeutic drug levels than under MMI medication [87]. A possible explanation could stem from intrinsic microbial enzymatic activity. Noticeably, some bacterial strains possess trimethylamine monooxygenase, which can metabolize PTU the same way as liver flavin-dependent monooxygenase (FMO3) [18]. Yan et al. [88] reported that *Prevotella* might also affect the therapeutic efficacy of drugs for GD. Glucocorticoids and immunosuppressive drugs (azathioprine and mycophenolate) also used in the treatment of GO are known to affect microbiota composition, however data regarding specifically GO patients are lacking [34].

### Thyroid cancer

It is accepted that dysbiosis has a carcinogenic effect on gastrointestinal cells, but its role on extraintestinal ones still needs to be ascertained. However, carcinogenesis relies mainly on two mechanisms: DNA damage and cellular apoptosis, on one side, inflammatory reactions and immune surveillance on tumor growth, on the other [25, 40]

In patients with TC or thyroid nodules, one study [89] showed an increase in gut microbial richness and diversity compared to healthy controls. Specifically, at phyla level, Firmicutes were increased in stool sample of TC patients, with an increase in *Streptococcus* and reduction in *Butyricimonas* and *Lactobacillus* [89]. Reduction of *Butyricimonas* and *Lactobacillus* can affect some products of bacterial metabolism, such as SCFAs and especially butyrate, important for its immunoregulatory effect. Therefore, their reduction could lead to increased cellular proliferation, and ultimately to a higher risk of cancer [89]. Another study [90] instead, showed a reduction in microbial richness, especially of the butyrate-producing gut microbiota, both in TC and in thyroid nodules with a high Thyroid Imaging Reporting and Data System (TI-RADS) score, meaning a higher ultrasound risk of being malignant. To note, in one of these cohorts [91], 2/3 of patients had lymph node metastases at diagnosis.

A further interesting finding is the existence of an intratumoral microbiota, mainly represented by intracellular bacteria found in cancer cells and peritumoral tissues. It has been shown that, in patients with thyroid cancer, tumoral and intestinal microbiota are different [92]. In detail, a prevalence of Proteobacteria was seen in thyroid samples, while Firmicutes were more represented in stools. Other studies [93, 94], instead, investigated the difference between tumoral and peritumoral microbiota composition. Results displayed a general lower microbic abundance in tumor tissue, associated with reduced richness and diversity indexes, while an increase in *Sphingomonas* was observed [94]. Noticeably, *Sphingomonas* abundance was higher in N1 stage compared to N0 stages [94]. Owing to these findings, *Sphingomonas* genus has been proposed as a marker to distinguish tumoral from peritumoral tissue and to suspect the presence of lymph node metastases [29, 37]. From a clinical point of view, thyroid microbioma appeared to be different between sexes and histologic tumor subtypes, and a strong positive correlation with MACIS score (distant Metastasis, patient Age, Completeness of resection, local Invasion, and tumor Size) was found for *Micrococcus luteus* and *Bradyrhizobium sp* [94]. GM could have an impact on treatment as well. Radioactive Iodine treatment (RAI) is a common tool in the post-operative management of TC to prevent or to treat recurrences, and iodine uptake by cancer cells is essential for its efficacy. GM can influence iodine uptake and could be associated to RAI-refractory papillary thyroid carcinoma through different mechanisms involving NIS and thyroglobulin expression as well as TSH levels [95]. Noticeably, an *in vitro* study demonstrated that SCFAs (butyric acid) can inhibit histone deacetylase, activating NIS re-expression in thyroid cancer cells and inducing iodine uptake and redifferentiation. The modulation of SCFAs production has been proposed as intriguing field of research, since they are thought to exert a regulatory effect also in immune microenvironment [41].

### Thyroid-related micronutrients and gut microbiota

**Fig. 3:**
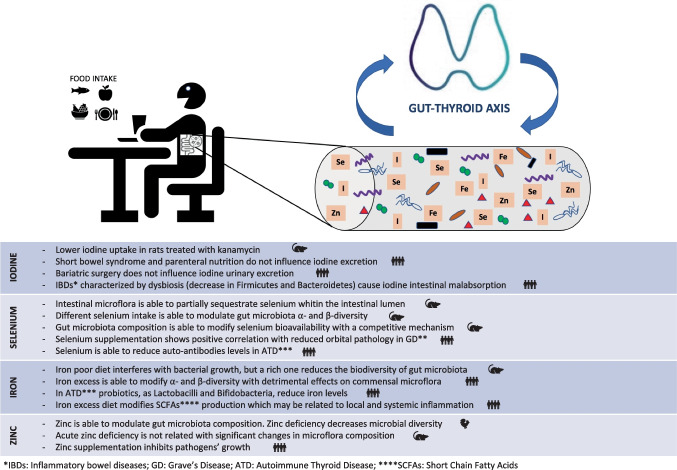
Main evidence of the bidirectional relationship between micronutrients homeostasis and gut microbiota composition obtained in murine models (  ), in chickens (  ) and in humans (  )

Intestinal barrier is a semi-permeable wall that allows the uptake of nutrients from the intestine being some of them key for normal thyroid functioning. In the following section we will discuss the interaction between gut microbiota and representing some of the main thyroid-related micronutrients (Fig. 3).

#### Iodine

Iodine is an obliged constituent of thyroid hormones’ structure and most of the iodine contained in the human body is stored in the thyroid [24]. Iodine uptake by thyroid is an active process and one of the limiting steps of thyroid hormones synthesis. The NIS, the widely diffuse iodine transporter, uses the flux obtained by the exchange by intracellular H^+^ with extracellular Na^+^ to co-transport iodine against its electrochemical gradient [96]. Also, iodine intestinal absorption is mediated by the NIS located in the apical part of plasma membrane of epithelial gastrointestinal tract. Further transporters responsible for intestinal iodine absorption are Na + /multivitamin transporters and cystic fibrosis transporter but to a lesser extent [22].

In 1972, a paper by Vought showed the role of gut microbiota in modulating iodine absorption in rats: animals treated with kanamycin, an antibiotic lowering total bacterial counts in rats, showed a reduced radioiodine uptake at 3 h and following 42 and 72 days of treatment, compared to conventional reared rats [97]. This evidence is in keeping with the results of the study by Nicola et al. demonstrating that LPS is able to increase NIS gene expression [61]. However, in human studies, the results are more conflicting. Indeed, in subjects with short bowel syndrome and in parenteral nutrition, iodine excretion was not significantly different from healthy controls, in spite of the different microbiota composition between the two groups [31, 44, 47]. Similar results were observed in iodine urinary excretion in post-bariatric patients by Michalaki et al. [98] However, in humans with inflammatory bowel diseases, a condition usually accompanied by dysbiosis, it was observed a condition of iodine malabsorption [18, 22]. Furthermore, it has been observed that the reduction in butyrate-producing gut microbiota is related to a reduction in iodine uptake and this evidence was associated with the pathogenesis of high-risk thyroid nodules [48]. Indeed SCFAs, especially butyric acid, through the inhibition of histone deacetylase, may activate NIS re-expression in thyroid cancer cells (see. Thyroid cancer section) [41].

On the other side, due to its intrinsic antimicrobial activity, iodine may alter gut microbiota composition, affecting both resident and pathogen bacteria [24, 31]. Indeed, it has been clearly demonstrated that iodine may interfere with the electron chain transport, by inhibiting ATP production in aerobic bacteria; its ability to disrupt microorganism cell wall structures has also been described [99, 100]. The effect of iodine supplementation has been analyzed in some animal models indicating that the overall effect of iodine on microbiota modulation would depend on the individual condition of host microbial composition [101, 102].

#### Selenium

Selenium concentration in the thyroid is higher than in any other organ [103]. It is an essential constituent of a group of proteins known as selenoproteins that are involved in several processes, the more important being the antioxidant and anti-inflammatory actions as well as the metabolic activity on thyroid hormones [103]. In nature, selenium exists in inorganic forms, selenate and selenite, and in organic ones, as a sulfur amino acid analog, selenomethionine and selenocysteine [30]. The absorption of these compounds occurs in duodenum, cecum and colon, being the absorption of organic forms quicker than the inorganic forms’ one.

Selenium and gut microbiota composition interact each other. Indeed about 25% of all bacteria possesses genes encoding selenoproteins: *Escherichia coli*, for example, possesses three selenoproteins in its structure [104]. Some species of *Lactobacillus*, are able to convert intracellular selenite into the organic forms, facilitating selenium absorption in human body [30]. A study on germ-free as compared to normally reared mice suggested a partial sequestration of selenium by intestinal microbiota [105]. This kind of competition with the host for selenium uptake is mostly evident in condition of limited selenium availability [104]. Moreover, a different intake of selenium is able to modulate the composition of gut microbial environment: compared to mice fed with a selenium-deficient diet, animals with supranutritional supplementation showed a lower relative abundance of *Dorea* and an increased abundance of *Turicibacter,* that exerts antinflammatory activity in the gut, and *Akkermansia*, that is known for its protective effect in intestinal barrier integrity [106]. To note, *Akkermansia* showed a positive correlation with reduced orbital pathology in a murine model of GO [76]. A double-blind randomized clinical trial carried out in 2011 showed that selenite supplementation improves the ocular outcome in patients with mild GO [107]. The recommendation of a 6-month selenium supplementation to patients with mild and active GO of recent onset is present in the 2021 EUGOGO guidelines [108]. Indeed, selenium exerts a beneficial activity on immune system modulation, also promoting Tregs cytokines secretion [41]. Frequently, a selenium deficit is described in Hashimoto’s thyroiditis and the administration of selenium seems to be able to reduce thyroid autoantibodies levels [30]. In patients affected by Hashimoto’s thyroiditis not replaced with levothyroxine, it was observed an increase in some *Lactobacillus* species, that are positively associated with selenium levels [30, 40].

#### Iron

Iron plays a key role in thyroid function because of TPO enzyme contains iron in the active center and is also involved in the storage and secretion of thyroid hormones [22]. Iron deficiency has a deep impact on thyroid metabolism because when anemia occurs it may lower oxygen transport, inducing a condition resembling the thyroid impairment of hypoxia [18, 22]. In particular, animal models of iron deficiency show a thyroid functions impairment [22]. The uptake of all forms of iron (inorganic, heme, and ferritin) occurs mainly in the duodenum and upper jejunum [18]. Iron is absorbed in the reduced form of Fe (II) and the efficiency of colonic iron absorption is only about 15% as compared the one occurring in the duodenum [109, 110]. However, this percentage may be modulated by the pH variation in the colon that may be caused by SCFAs production [111]. Moreover, it has been demonstrated that *Lactobacillus fermentum* shows ferric-reducing activity, enabling Fe(III) to Fe(II) reduction, thus facilitating iron absorption [31, 109]. Indeed, bacteria are able to modulate iron bioavailability owed to several high-affinity proteins facilitating its uptake. Therefore, as observed for selenium, gut microbiota and the host compete for iron absorption [18, 31]. Animal studies observed that an iron poor diet interferes with bacterial growth, while a rich one reduces the biodiversity of gut microbiota [18]. In humans, iron supplementation increases Enterobacteriaceae and Bacteroidetes while decreases Lactobacillaceae and *Bifidobacteria*. To note, these latter, require no iron to grow [1, 18]. The competition for unabsorbed iron also modulates the microbiota composition with detrimental effects for commensals. A heme rich gut environment provides nutrients for the proliferation of bacterial species that are able to metabolize this compound. Notably, an excessive intake of iron increases pathogenic intestinal bacteria (*Salmonella*, *Shigella*, pathogenic *E. coli*) which require iron for their colonization and virulence [22, 109]. Likewise, in humans exposed to iron excess diet, the modification of SCFAs production which may be observed.

#### Zinc

The role of zinc in thyroid pathophysiology is due to its involvement in both deiodinase and superoxide dismutase activities. Furthermore, it is a component in thyroid hormone binding transcription factor [22, 112]. Indeed, zinc is involved in the synthesis of the chief components of the whole thyroid machinery [thyrotropin-releasing hormone (TRH), TSH and of thyroid hormones]. Beyond that, it may influence the triiodothyronine binding with its nuclear receptor [22, 46]. In humans, it has been observed a reciprocal relationship between thyroid disorder and zinc metabolism since hypothyroid patients often present reduced levels of zinc as well as zinc deficiency correlates with low level of free thyroid hormones [46].

Some animal studies, conducted on mice and chicken, described a correlation between gut microbiota composition and zinc. Human evidence supported the effect of zinc supplementation in inhibiting the growth of pathogens (i.e., diarrhea pathogenic *E. coli*-related), promoting the growth of beneficial bacteria such as *Lactobacillus* [18, 113]. Furthermore, in human affected by thyroid autoimmune diseases, the relative abundance of *Lactobacillus* and *Bifidobacterium*, positively correlate with zinc levels [40].

### Nutritional management of thyroid disorders

It has been clearly demonstrated that dietary habit is one of the major determinants of gut microbiota composition [114]. In some of the selected reviews [15, 19, 34, 39, 43] the possible interplay between diet, GM and thyroid autoimmunity are reported. Some studies detected significant differences between the dietary habits in patients with Hashimoto’s thyroiditis compared to healthy subjects. One of them [66] tried to correlate HT, diet and gut microbial composition. However, despite the observed difference in microbiota composition, it was difficult to discriminate the contribution of diet and/or of disease to the microbial signature of patients. A beneficial effect of gluten free diet has been proposed in patients with Hashimoto’s thyroiditis [115]: the rationale of this proposal came from the partially shared genetic background of these two autoimmune disorders and the frequent co-presence in the same subject. However, the combined results of the trials published on this topic does not justify the use of this elimination diet in patients with Hashimoto’s thyroiditis [116]. Furthermore, this diet, frequently characterized by low complex carbohydrate and fiber as well as a high saturated fatty acids and sugar intakes, is often accompanied by deficiencies in iron, calcium, magnesium, vitamin D, E and some of group B [117]. Due to the multiple deficit in micronutrients and vitamins sometimes detected in patients with Hashimoto’s thyroiditis, its inflammatory nature and the dysbiosis described in this disorder, Inhatowitz et al. [19] suggested a nutritional approach. This diet is based on the following principles: to consume an adequate amount of proteins; to increase the intake of polyunsaturated fatty acids (particularly omega -3) while lowering the one of saturated fatty acids, due to their potential conducive to gut leakiness; to choose products with lower degree of processing; to consume an adequate amount of fibers that may properly nourish gut microbiota; to measure, and in case of deficiency, to supplement micronutrients and vitamins. Furthermore, Inhatowitz et al. [19] emphasized the possible coexistence, in patients with thyroid autoimmunity, of concomitant gastrointestinal autoimmune comorbidities: these diseases, such as celiac disease, gastric atrophy or inflammatory bowel disorders, may exacerbate micronutrients and vitamins deficiency [118].

As far as concern with Graves’ disease patients, a paper described a lower risk of thyroid hyperfunction in people following lacto-ovo and pesco-vegetarian diets compared to omnivores [119]. Noteworthy, several papers examined the beneficial effect of Mediterranean diet in several pathological conditions (type 2 diabetes mellitus, cardiovascular disorders and some type of cancer). From a gut microbial point of view, it is characterized by higher abundance of Bacteriodetes and *Prevotella* and by a lower concentration of Firmicutes. Studies on GD’s microbiota composition often described an increased abundance of *Prevotella,* and it has been hypothesized that Mediterranean diet might not be a good choice for these patients [34]. However, most of the data about gut microbiota composition were obtained analyzing Asian populations, preventing to draw clear conclusion on the effect of this diet in GD/GO patients [34]. This field remains fully open to novel information.

### Probiotics, prebiotics and synbiotics

The term probiotics has been introduced in the early 70’s to define live microorganisms that, given in the appropriate amount, exert beneficial effects on the host’s health [120]. In the last years, other compounds have been added to probiotics as over-the-counter products such as “prebiotics”, that are “substrates selectively utilized by host microorganisms conferring a health benefit” [121], and “synbiotics”, that are a mixture of pro- and prebiotics [122]. Probiotics are among the most consumed food supplements worldwide and may be produced as enrichment for food or as lyophilized compounds, commercialized in granulated or in pills formulations [123]. Despite the general popularity of these products, the indications and the actual benefit of their use are not always clear and universally supported. Despite some animal studies described a thyroid function benefit derived from probiotic supplementation [124], only few studies examined the effect of their use in patients with thyroid disorders. The recent systematic review with meta-analysis by Zawadzka et al. [49] included the RCTs [125–128] dealing with the effect of 8 weeks probiotic or symbiotic supplementation in hypothyroid patients treated with levothyroxine. All the studies reported lower TSH values in supplemented patients than in control ones, without reaching statistical significance. Patients supplemented with synbiontic experienced a lower severity of constipation while the other symptoms were similar in supplemented and un-supplemented patients. To note, in the study by Spaggiari et al. [125] a lower need for LT4 dose adjustments has been described in supplemented patients and, in one by Talebi et al. [128], a slightly reduced LT4 requirement in the synbiotic-treated patients has been detected. However, these small variations and the low number of patients, prevented the authors to give a clear result about this topic. Furthermore, these studies lack characterization, before and after supplementation, of patients’ microbiota. The overall results of these RCT indicated that routine administration of probiotics and/or prebiotics should not be recommended to patients with primary hypothyroidism.

Three further studies analyzed the effect of probiotic and prebiotic supplementation in Graves’ disease patients treated with methimazole. In particular, the coadministration of methimazole and *Bifidobacterium longum* led to an improvement of thyroid hormones levels and a drop in TRAb levels to the normal ones in 9 patients; this last effect was not observed in the 8 patients treated with MMI alone [129]. A further study [130], examined the effect of the co-treatment with methimazole and berberine, a natural alkaloid, suggesting a role for it in modulating gut microbiota in 10 patients with GD. A randomized trial on the use of LAB4 probiotic (a mixture of two *Lactobacillus acidophilus* strains Plus *Bifidobacterium bifidum* and *Bifidobacterium animalis*) demonstrated the ability of probiotic mixture to modify microbiota composition in GD patients. It was shown a significant reduction of Firmicutes abundance and a transient decrease of IgA and IgG serum concentration without a clear effect on TRAb levels as well as on the relapse rate of the disease. In an animal model of GD, the administration of LAB4 promoted the induction of GD/GO phenotypes, in spite of increased orbital concentration of Treg lymphocytes [76]. These result, also considering the increased relative abundance of Lactobacillus that characterized patients with both GD and GO as well as the demonstration that specific probiotic strains are able, in experimental settings, to exacerbate different autoimmune disorders [12], seem to suggest that, in supplementing these patients, probiotic formula should not contain Lactobacillus strains [34].

A RCT published in 2022 [131] investigated the role of a mixture of probiotic species in alleviating symptoms related to thyroid hormone withdrawal in thyroidectomized patients waiting for the administration of radioactive iodine treatment. In the 25 patients supplemented with probiotics, the lack of energy, constipation and dry mouth incidence as well as serum LPS and lipid values were lower as compared to the 25 un-supplemented patients.

### Gut microbiota transplantation

**Fig. 4:**
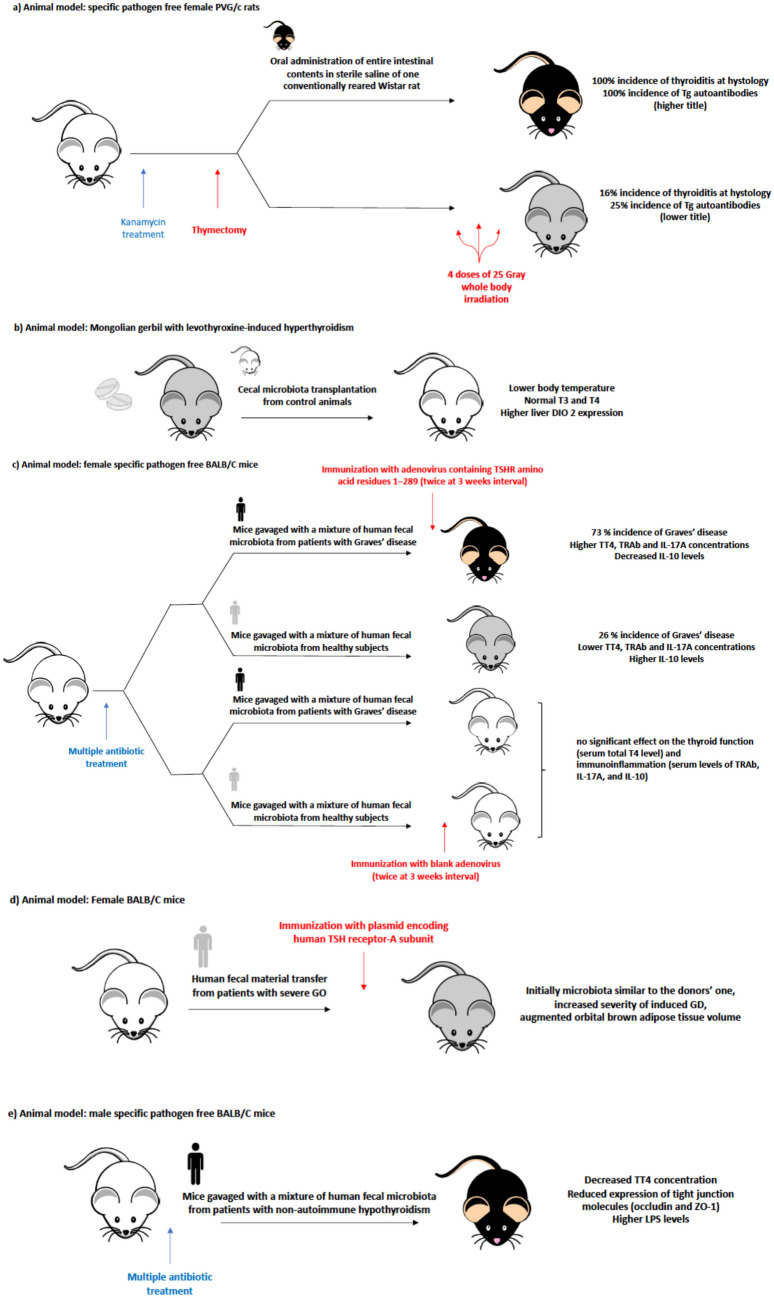
Designs and results of the experiments on Fecal Microbial Transplantation in murine models of thyroid disorders available in the literature: **a** ref. [62], **b** ref. [132], **c** ref. [133], **d** ref. [76], **e** ref. [67]

Only five papers cited in the 38 reviews included in this systematic review examined the effects of gut microbiota transplantation. In particular, animal models received in two experiments a transplantation from animal models’ stool, while in three of them, the animals were transplanted with human fecal content. In Fig. 4 are depicted the designs and the main results of the five studies on this topic. The first study on this topic was published in 1988 [62] and evaluated the role of intestinal flora in determining the susceptibility to autoimmune thyroiditis in female PVG/c strain rats. The authors noticed that rats belonging to the same species, that had been reared under specific pathogen-free (SPF) conditions, were significantly less susceptible to the induction of experimental autoimmune thyroiditis (by thymectomy and irradiation) than conventionally raised rats. The same authors treated a group of female PVG/c SPF rats with kanamycin and then thymectomized them. Afterward, they transplanted a fraction of them with the homogenized fecal content of a Wistar conventionally reared rat. Then, both the groups (transplanted and not) were irradiated with 4 doses of 25 Gray whole-body irradiation. The conventionalized group showed a higher incidence of experimental autoimmune thyroiditis compared to SPF rats (Fig. 4a). Another study examined the effect of cecal microbial transplantation in Mongolian gerbils [132]. In this experiment gerbils, that had become hyperthyroid due to previous levothyroxine treatment, were transplanted with the cecal microbiota from control animals by intragastric gavage. This procedure attenuated hyperthyroid thermogenesis, with a quicker recovery of body temperature and resting metabolic rate, increased type 2 deiodinase expression in the liver, and higher decline of both T3 and T4 levels compared to control animals, that had been treated only with intragastric saline solution (Fig. 4b).

The effect of fecal microbial transplantation from humans with GD to SPF female BALB/c mice has been analyzed [133]. Following treatment with multiple antibiotics, the authors transplanted homogenized fecal flora from patients with GD and from healthy subjects to the murine samples, confirming through PCoA analysis that the transplant was successful. Then they transfected a part of the animal sample with the adenovirus containing TSHR amino acid residues 1–289; the rest of the sample was transfected with the blank adenovirus vector**,** which exerted no significant effect on thyroid function and immuno-inflammation in the mice (Fig. 4c). The group of mice transfected with the adenovirus containing TSHR amino acid residues 1–289 showed higher total thyroxine, TRAb and interleukin-17A levels and lower levels of IL-10. However, some differences appeared depending on the source of the microbiota transplantation they received: in the mice group with transplantation from GD patients, the incidence of Graves’ disease was 73% compared to the one of mice transplanted with fecal content of healthy subjects that was 26%. The functional and immuno-inflammation markers were also significantly different in the two groups. The authors concluded that a dysbiotic state alone may not be sufficient to trigger Graves’ disease, but it may be a key factor together with other pathogenic events in determining the onset and the course of this disorder (Fig. 4c).

A further paper examined the effect of transplantation from human donors bearing Graves’ sight-threatening ophthalmopathy to an animal model recipient constituted by female BALB/c mice [76]. The animals were immunized with the human thyrotropin receptor (hTSHR)-A subunit (hTSHR289). The human fecal material transfer caused a variation in mice’s microbiota composition that initially was similar to the donors’ one, an increased severity of induced GD and an augmented orbital brown adipose tissue volume (Fig. 4d).

The last paper, on the contrary, analyzed the effect of transplanted human fecal microbiota from patients with non-autoimmune primary hypothyroidism to pathogen-free BALB/c male mice [67]. Following multiple antibiotic treatments, mice transplanted showed decreased TT4 concentration, reduced expression of tight junction molecules (occludin and ZO-1) as well as higher circulating LPS levels. On the contrary, fecal SCFAs, such as butyrate and acetate, concentration was reduced (Fig. 4e).

## Conclusions

The systematic analysis of these reviews confirms the existence of the gut-thyroid axis, due to several evidence of mutual interference of these two systems. However, the identification of a causal link between the variations of the intestinal microbiota composition and the pathogenesis of the most common thyroid diseases is still far from being demonstrated. Until now, in fact, the studies have been conducted above all in Asia and in a small number of patients and not always taking into account the numerous environmental and personal elements (pollution, ethnicity, dietary habits, lifestyle, drug intake) that exert a key role in shaping gastrointestinal microenvironment. Further large-scale studies, involving different ethnic groups and areas of the world, are necessary in order to identify whether specific microbial signatures of thyroid disorders do exist. This analysis will then have to be integrated by metagenomic studies that can identify altered metabolic pathways in patients with different thyroid diseases. The identification of specific microbial and metabolomic profiles must be a prerequisite for the rationale and targeted use of the microbiota modulating agents (diet, probiotics and microbiota transplantation) for preventive and therapeutic purposes of the most common thyroid disorders.

## Abbreviations

CAS: Clinical activity score
CYP7A1: Cholesterol 7α-hydroxylase
GALT: Gut-associated lymphoid tissue
GD: Graves’disease
LPS: Lipopolysaccharide
GF: Germ-free
GO: Graves’ ophthalmopathy
IBD: Inflammatory bowel disorders
MACIS: Distant Metastasis, patient Age, Completeness of resection, local Invasion and tumor Size
MAPK: Mitogen-activated protein kinase
MDI: Microbial dysbiosis index
MMI: Methimazole
NAFLD: Nonalcoholic fatty liver disease
NIS: N^+^/I^−^ symporter
NLRP3: NLR family pyrin domain containing 3;
PTU: Propylthiouracil
RAI: Radioactive iodine treatment
SCFA: Short-chain fatty acid
SFP: Specific pathogen-free
TC: Thyroid cancer
Tg: Thyroglobulin
TGR5: Takeda G-protein coupled receptor 5
TIRADS: Thyroid Imaging Reporting and Data System
TLR: Toll-like receptor
T3: Triiodothyronine
TPOAb: Anti-thyroperoxidase autoantibodies
